# Social Mismatch and Affective Wellbeing: An Ecological Momentary Assessment Study

**DOI:** 10.1007/s10902-025-00965-6

**Published:** 2025-10-23

**Authors:** Lianne P. de Vries, Meike Bartels

**Affiliations:** 1 Department of Biological Psychology, Vrije Universiteit Amsterdam, Van der Boechorststraat 7, 1081 BT Amsterdam, The Netherlands; 2 Amsterdam Public Health Research Institute, Amsterdam University Medical Centres, Amsterdam, The Netherlands

**Keywords:** Positive and negative affect, Ecological momentary assessment, Social contact, Social mismatch, Wellbeing, Depressive symptoms, Genetic predisposition

## Abstract

The affective benefits of social contact depend on momentary social needs. Mismatch between desired and actual contact can result in social deprivation or oversatiation. We examined these dynamics in relation to affective wellbeing, considering genetic predispositions for loneliness, wellbeing, and depressive symptoms. A general-population sample of 1,086 adults (71% female, M_age_ = 36.4) completed 7-day Ecological Momentary Assessments of affect and social contact eight times daily. Social mismatch was higher when alone (M = 3.67) than with others (M = 2.65). Dynamic Structural Equation Modelling showed higher negative affect (β = .18 and β = .04) and lower positive affect (β = -.19 and β = -.05) was related to more social mismatch concurrently and two hours later. Unexpectedly, social oversatiation was linked to *reduced* negative affect two hours later (β = -.03). Younger adults and those with a stronger genetic predisposition for wellbeing showed stronger effects of social mismatch on positive affect. The results highlights the complex relationship between social contact, needs, and affective wellbeing.

**Supplementary Information:**

The online version contains supplementary material available at 10.1007/s10902-025-00965-6.

## Introduction

Social contact can be a strong resilience factor for wellbeing and psychological and physical health (Pinquart & Sörensen, 2000; Ren et al., 2022; Rico-Uribe et al., 2018). An absence of social contact can result in feelings of loneliness and social isolation, which are strongly associated with mental health problems and lower wellbeing (Cacioppo & Patrick, 2008). However, benefits of social contact on momentary affective wellbeing, i.e., positive and negative affect, depend on the social needs of a person at that moment, i.e., the desire to be in contact with other people or to be alone (Krämer et al., 2023). Such social needs differ both between persons and within persons over time (Coplan et al., 2019; Lay et al., 2019; Wrzus et al., 2024). Here, we investigated the between- and within-person relations between social contact, social needs, and affective wellbeing in daily life, including influences of genetic predispositions for loneliness, wellbeing and depressive symptoms.

To investigate behaviour and experiences in real-time and the daily context of people, Ecological Momentary Assessment (EMA) can be used where participants answer multiple small surveys per day or week (Stone & Shiffman, 1994). EMA studies have shown, on average, a direct affective benefit from social interactions, i.e., people experience greater positive affect and less negative affect in social contact compared to being alone (Bernstein et al., 2018; Liu et al., 2019; Sun et al., 2020). However, the affective benefit from social contact can differ from moment-to-moment within persons. Individuals can experience a mismatch between their desire for social contact and their actual interactions, resulting in less positive or even negative effects of social contact on affective wellbeing. This social mismatch can either result from *social deprivation*, i.e., absence of desired social contact, or *social oversatiation*, i.e., experience of unwanted company or social contact (Wrzus et al., 2024). Thus, states of social mismatch indicate an unpleasant social situation or social stress (Reininghaus et al., 2016). Krämer et al. (2023) investigated real-time dynamics of social contact, social desire, and momentary affect on two weekend days. On average, social oversatiation was associated with less positive affect and more negative affect, while social deprivation during the weekend was not related to momentary affect. Associations during week days are unknown.

The associations between social dynamics and affect can depend on the time scale at which they are examined. The lagged effects of social mismatch on affect, i.e., minutes to hours later, might be different compared to the concurrent associations. Theory on stress and adaptation (Lazarus & Folkman, 1984) suggests that appraisal and coping processes occur within minutes to a few hours, creating short-term carry-over effects distinct from momentary reactions. Therefore, we examined both concurrent and 2-h lagged associations between social mismatch and affect.

Furthermore, the effects of social stress on affective wellbeing can differ between persons. Individuals differ in their affective reactivity to social stress (Leger et al., 2018). Some people are sensitive to social stress and social mismatch has a large effect on their positive and negative affect, whereas others are less sensitive. These differences are expected to be partly explained by individual characteristics and genetic predispositions (Monninger et al., 2022). Based on known associations with social contact, we expect that feelings of loneliness, overall wellbeing, and depressive symptoms, as well as genetic predisposition for these traits will influence the sensitivity to the social environment and thus the effect of social mismatch on wellbeing.

*Loneliness* can be defined as *the unpleasant experience that occurs when a person's network of social relations is deficient in some important way, either quantitatively or qualitatively”* (Perlman & Peplau, 1981)*.* Loneliness is thus the perception of the discrepancy between actual and desired level of social interactions (Wheeler et al., 1983). *Wellbeing* is broadly defined as feeling good and functioning well (van de Weijer et al., 2022; Lomas et al., 2022). Social contact is strongly related to wellbeing or even a component of wellbeing (Keyes, 1998; Lambert et al., 2020). Individuals with a higher general wellbeing have more high-quality social interactions, and higher positive affect and lower negative affect in daily life (de Vries & Bartels, 2025; Houben et al., 2015). *Depressive symptoms* include persistent sadness or hopelessness, loss of interest or pleasure, and fatigue or loss of energy (American Psychiatric Association 2013). High-quality social contact is protective for the development of depressive symptoms (Gariépy et al., 2016; Li et al., 2023). Furthermore, individuals with more depressive symptoms react more strongly to stressors (Booij et al., 2018; Cohen et al., 2005).

Finally, *genes* play a significant role in mental health, wellbeing, and (preferences for) social contact (Okbay et al., 2016; Day et al. 2018; Baselmans et al., 2019; Howard et al., 2019; Bralten et al., 2021; Socrates et al., 2023; Flint, 2023). Genetic predispositions for these individual characteristics can be summarized into a polygenic score (PGS). PGSs are a measure of an individual's genetic probability to develop a certain disorder or have a certain trait (Wray et al., 2007). These previous findings suggests that (genetic predisposition for) loneliness, wellbeing, and/or depressive symptoms influence the experience of social mismatch and the associations between social mismatch and affect.

To summarize, we investigated the within- and between-person effects in temporal dynamics between social mismatch and affect using Dynamic Structural Equation Modelling (DSEM). We related person-specific estimates to survey data and polygenic scores for loneliness, depressive symptoms, and wellbeing. We expected more social mismatch to be related to less positive affect and more negative affect at the same moment and two hours later. In addition, we expect higher loneliness, depressive symptoms, and lower wellbeing, as well as genetic predisposition to these traits to be associated with more affective reactivity, i.e., stronger decreases in positive affect and stronger increases in negative affect, to social deprivation and social oversatiation. Finally, we explore the separate effects of social deprivation and social oversatiation to investigate differences of lack of contact versus unwanted contact on affect. The study design, hypotheses, and planned analyses with code were preregistered on https://osf.io/r2x3u.

## Methods

### Participants

Participants are voluntarily registered at the Netherlands Twin Register (NTR) (Ligthart et al., 2019). The NTR sample is a population-based, non-clinical sample. Every two/three years, longitudinal survey data about lifestyle, personality, psychopathology, and wellbeing are collected in twins and their families. DNA data have been collected for a subsample of participants. Regularly, subsamples of participants are invited to participate in different non-survey projects, such as the recent smartphone-based EMA study. The data of the EMA study were collected between September 2022 and April 2023. To be able to participate in the EMA study, participants had to be healthy adults of 18 year or older, a monozygotic or dizygotic twin, and to have a smartphone with internet access at least once a day. Participants with a physical and/or mental disorder diagnosed by a doctor or having a physical or mental disorder that hinders them in daily life could not participate.

In total, 1165 participants took part in the study and downloaded the smartphone application. Of these participants, 1086 participants completed at least one EMA survey and were included in the analyses. Participants were on average 36.4 (SD = 12.5) years old and the majority was female (71%, see Table 1). For a subset of 671 participants, survey data on loneliness, depressive symptoms, and wellbeing was available to be included in the regression models. Finally, including only participants with both survey and PGS on loneliness, depressive data, and wellbeing resulted in a subsample of 556 participants.

**Table 1:** Descriptives

	Mean	SD	min	max	ICC	Total n
Sex (% females)	71%				–	1086
Age during EMA	36.6	12.5	18	64	–	1086
Nr of beeps completed	35.7	14.17	1	56	–	1086
% of beeps alone	0.37	0.21	0	1	–	1086
% of beeps with others	0.63	0.21	0	1	–	1086
PA when alone	6.93	1.13	3.36	10	0.56	1052
PA when with others	7.18	1.04	3.75	10	0.53	1078
PA overall	7.08	1.05	3.56	10	0.53	1086
NA when alone	2.32	1.08	1	6.61	0.61	1051
NA when with others	2.22	0.99	1	6.08	0.59	1077
NA overall	2.27	1	1	6.24	0.59	1085
Social deprivation	3.67	1.86	1	10	0.50	1052
Social oversatiation	2.65	1.33	1	9	0.36	1078
Social mismatch overall	3.01	1.22	1	9	0.24	1086
Loneliness	3.94	1.22	3	9	–	718
Depressive symptoms	4.00	3.88	0	28	–	698
Wellbeing	27.26	5.18	6	35	–	720

### Procedure Smartphone-Based EMA Study

The EMA study has been approved by the Medical Ethical Committee at the VU medical center (METc VUmc; 2021.0716). Participants of the Netherlands Twin Register were invited by email to participate in the EMA study. After providing informed consent, participants received an email with instructions how to install the *NTR welbevinden* app (developed by Ethica Data Sevices Inc, 2019 under a data agreement to comply with GDPR regulations) on their own device through which they received the questionnaires. Notifications were programmed locally on participants’ phones to enable the completion of questionnaires offline.

The study period of seven days started immediately after installing the app. Participants were asked to complete eight short questionnaires per day, including items of their momentary positive and negative affect and social environment (i.e., 18 items, ~ 2 min). The prompt design was semi-random, based on pre-specified blocks during the day and the waking hours of the participants. Participants were signaled (with buzzing or sound based on the settings of the participant’s phone) with the request to answer the questionnaire. After 30 min, participants received an automatic reminder if the questionnaire was not completed. After 60 min, the questionnaire expired. Participants did not receive payment, but they did receive a personal rapport on their own wellbeing during the week, and influence of contextual factors on wellbeing, like location (inside vs outside), company they were in (partner, children, colleagues, friends etc.), and activity they were doing (working, free time active, free time passive, etc.).

### Measures

We used survey data of the Netherlands Twin Register for loneliness, wellbeing, and depressive symptoms. These traits were assessed in different NTR surveys (Ligthart et al., 2019). If data were available in multiple surveys, we used data collected closest to the EMA data collection.

#### Loneliness

 Loneliness was assessed using the short scale for loneliness (Distel et al., 2010; Hughes et al., 2004). The scale includes 3 items from the R-UCLA loneliness scale. Participants rate how often they identify with the items on a scale from 1 to 3 (1 = hardly ever, 2 = some of the time, 3 = often). An example item is “*I feel isolated*”. Answers were summed to obtain a sum score with higher score representing higher levels of loneliness. Internal consistency is good with α = 0.72 (Hughes et al., 2004).

#### Wellbeing

Wellbeing was assessed using the Satisfaction with Life scale (Diener et al., 1985). The Satisfaction with Life scale has five items to report life satisfaction on a 7-point Likert scale, ranging from 1= strongly disagree to 7 = strongly agree. An example item is ‘*In most ways my life is close to ideal’*. Internal consistency of the scale in the current sample is good with α = 0.87.

#### Anxious-Depressed Symptoms

Symptoms of anxiety and depression were assessed with the Anxious-Depressed subscale of the ASEBA Adult Self Report (Achenbach & Rescorla, 2003). This subscale consists of 13 items and participants rate the occurrence of the behavior, now or within the past 6 months, on a 3-point scale (0—not true, 1—sometimes true, and 2 -very true or often true). An example item is “*I am unhappy, sad, or depressed*”. The anxious-depressed score was computed by summing the items, with a higher score reflecting more anxious-depressed symptoms. Internal consistency of the scale is good with α = 0.88 (Achenbach & Rescorla, 2003).

#### Polygenic Scores

To capture individuals’ genetic predispositions, we created *polygenic scores* (PGS) for loneliness, wellbeing, and depression. A PGS is a single summary number that reflects a person’s inherited tendency toward a trait, based on thousands of genetic variants identified in large genome-wide association studies (GWAS) (Wray et al., 2007). We used summary statistics of recent GWASs on loneliness (Neale Lab database, 2020), wellbeing (Baselmans et al., 2019), and depression (Major Depressive Disorder GWAS, Howard et al., 2019). For each participant, we summed their genetic variants across the genome, weighting each by its effect size from the GWAS, to obtain a PGS. These scores do not determine whether someone will have a trait but indicate a higher or lower genetic likelihood on average. PGS have been shown to predict a modest proportion of variance in psychological traits (typically up to one or a few percent, depending on the GWAS sample size and trait) and to reveal shared genetic influences between traits (Purcell et al., 2009). In this study, we used the PGS to examine whether genetic predispositions for loneliness, wellbeing, and depression relate to the strength of the associations between social dynamics and affective wellbeing.

### Ecological Momentary Assessment

#### Momentary affective wellbeing

Momentary affective wellbeing was assessed as positive and negative affect with items of the Positive and Negative Affect Scale (PANAS) (Watson et al., 1988). Items included were happy (Dutch: *gelukkig*), relaxed (*ontspannen*), energetic (*energiek*), and satisfied (*tevreden*) for positive affect and stressed (*gestressed*), afraid (*angstig*), irritated (*geïrriteerd*) and down (*somber*) for negative affect. Participants rated to what extent they felt these emotions in that moment on a visual analogue scale from 1 (not at all)—10 (very much). The selected items were chosen based on previous EMA work (e.g., Bülow et al., 2020; Houben et al., 2015; Kuppens & Verduyn, 2017). The positive affect items were averaged in a positive affect score per EMA assessment, and the negative affect items in a negative affect score. From a multilevel perspective, the overall composite reliability was .80 and .76 for positive and negative affect respectively, the between-person reliability (ability to detect differences across people) was .83 and .80, and the within-person reliability (ability to detect changes over time within person) was .74 and .68 (Lai, 2021).

#### Social Context and Mismatch

Social context was assessed with the question “With who are you?”. Participants could indicate multiple options, including “*Alone”, “Alone with strangers”, “Your partner”, “Children”, “Other family”, “Colleagues/classmates”, “Clients/customers”, “Friends”*, and “*Other people you know”*. When participants indicated to be alone, they were asked to indicate to what extent they rather would be with other people on a scale from 1 (*not at all*) to 10 (*very much*). A higher score indicates a desire to be with other people and thus more social deprivation. When participants indicated to be with others, they were asked to indicate to what extent they rather would be alone on a scale from 1 (*not at all*) to 10 (*very much*). A higher score indicates a desire to be alone and thus more social oversatiation. Both scores reflect the degree of social mismatch on a continuous scale from 1 (no social mismatch) to 10 (large mismatch), reflecting social stress (Vaessen et al., 2023). At each assessment, participants answered thus either (a) how much they would rather be with others (if they were alone) or (b) how much they would rather be alone (if they were with others). We used these context-specific responses to create a ‘social mismatch’ score. In addition, we used social deprivation (when alone) and social oversatiation (when with others) separately in different models to explore the differences between the different forms of social mismatch.

### Statistical Analyses

Analyses were preregistered at https://osf.io/r2x3u and code of the statistical analyses can be found at https://osf.io/drtah. Data of this and other NTR studies can be requested via https://ntr-data-request.psy.vu.nl/.

#### Descriptives and Correlations

We computed descriptive statistics (mean, standard deviation, ICC) and bivariate (within- and between-person) correlations for positive and negative affect, social deprivation, social oversatiation, and social mismatch overall. The intra class coefficient (ICC) indicates the part of variance that is due to between-person and within-person variation. Values closer to 1 suggest that most variance is between individuals, whereas values closer to 0 suggest that variance is largely due to within-person fluctuations and/or measurement error.

#### Dynamic Structural Equation Modelling

To test the dynamic relations between momentary positive or negative affect and social mismatch, we applied Dynamic Structural Equation Modelling (DSEM; Asparouhov et al., 2018; Hamaker et al., 2018) in MPlus (version 8.7). DSEM combines time series modelling and multilevel modelling to model individual differences in dynamic relations between the variables of interest within a person over time. We applied two main models, including within-person centred positive or negative affect in combination with social mismatch overall. In addition, we run four additional exploratory models, including positive or negative affect in relation to social deprivation or social oversatiation separately.

**Fig. 1:**
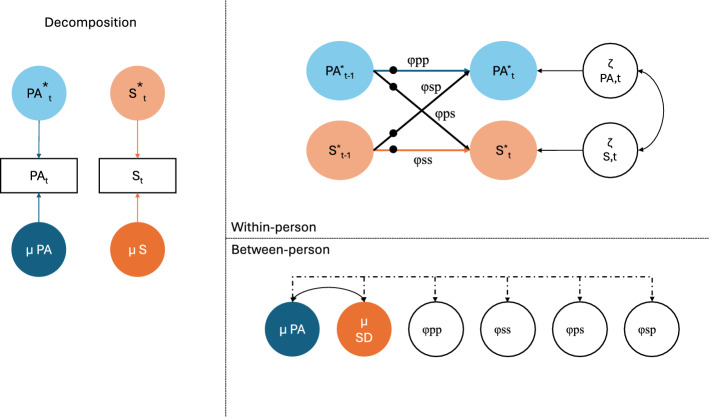
Multilevel dynamic structural equation model estimating the dynamic coupling between within-person centered momentary positive/negative affect and social mismatch. * represents within-person estimates. Black dots indicate random effects. µ = Means, PA = positive affect, S = social mismatch. φ indicates the (cross-)lagged effects, P-S indicates the concurrent within-person effects

In the DSEM model, within-person effects and between person-effects between affect and social mismatch can be estimated, both concurrent and cross-lagged effects (see Fig. 1). Via the TINTERVAL option, DSEM accounts for unequal time intervals between measurements that arise due to the semi-random design of the EMS study and missing data. The time interval was set at two hours, the average time between EMA survey prompts. Due to the Kalman filter in DSEM, all available observations can be used in the analyses. In Fig. 1, the φpp and φss indicate the person-specific autoregressive effects of respectively positive affect and social deprivation on subsequent time points, i.e., from moment t_−1_ to moment t. Cross-lagged effects between affect and social mismatch are indicated by φsp and φps. We include correlations between the random intercepts of all factors (including affect, social mismatch, and the autoregressive and cross-lagged effects, φpp, φss, φsp and φps).

#### Individual Differences in Effects

To investigate between-person effects in the relation between social mismatch and positive and negative affect, we extracted the person-specific standardized concurrent and cross-lagged effects from the models, using the *MPlusAutomation* package in R (Hallquist & Wiley, 2018). We applied six regression analyses with the concurrent effect of positive or negative affect and social mismatch and all cross-lagged (φsp and φps) effects from affect to social mismatch and vice versa as outcomes. We included sex, age, loneliness, wellbeing, and depressive symptoms survey scores and the polygenic scores as predictors.. We included the the genotyping array and first 10 genomic principal components as covariates to adjust for population stratification, as these components capture major axes of genetic variation (ancestry) within the sample and help prevent confounding of genetic associations (Abdellaoui et al., 2013). We used GEE to correct for familial relations in all regressions (Minică et al., 2015). We used *α* =.05 criterion for determining significance. Multiple testing is corrected for using the Benjamini–Hochberg method per model (Benjamini & Hochberg, 1995).

## Results

### Descriptives

Descriptive statistics for EMA variables, survey measures, and genetic data are shown in Table 1. Because these measures were collected at different time points and not all participants provided DNA or completed the survey, sample sizes vary across variables. The within- and between-person correlations between variables can be found in Table 2. Negative affect was higher when participants were alone (mean = 2.32) compared to when they were with others (mean = 2.22, t = 6.88, p <.001, d = 0.21). Similarly, positive affect was lower when alone (mean = 6.93) compared to when with others (mean = 7.18, t = −14.173, p < 001, d = 0.44). Furthermore, on average, participants indicated a low level of social mismatch (M = 3.01 on a scale of 0–10, SD = 1.22). Social mismatch was stronger when alone (i.e., social deprivation, M = 3.67), compared to when they were with others (i.e., social oversatiation, M = 2.65, t = 14.0, p <.001, d = 0.43).

**Table 2:** Correlations between the variables: within-person/between-person correlations

	PA	NA	Social deprivation	Social oversatiation	Social mismatch	% alone vs others	Lon	Depr	WB
Positive affect (PA)	1								
Negative affect (NA)	**−0.54/−0.71**	1							
Social deprivation	−0.01/−0.08	**0.04/.14**	1						
Social oversatiation	**−0.35/−0.44**	**0.32/.45**	−0.04	1					
Social mismatch	**−0.23/−0.41**	**0.20/.44**	**0.55**	**0.71**	1				
% alone vs others	**−0.22**	**0.17**	0.06	**0.15**	**0.32**	1			
Loneliness	**−0.29**	**0.24**	0.04	**0.17**	**0.17**	**0.15**	1		
Depressive symptoms	**−0.41**	**0.37**	0.03	**0.22**	**0.20**	**0.16**	**0.51**	1	
Wellbeing	**0.30**	**−0.27**	−0.03	**−0.24**	**−0.18**	**−0.12**	**−0.43**	**−0.53**	1

*Note* Bold correlations indicate significant correlations

The ICCs for positive and negative affect were between .53 and .61, indicating that around half of the variance is attributable to differences between individuals, with the remaining variance reflecting within-person fluctuations over time. This indicates that positive and negative affect are thus not stable but vary according to context, time, or situation. Similarly, the ICC for social deprivation was .50, while the ICC for social oversatiation was lower at .36, indicating that a greater proportion of the variance in oversatiation is driven by within-person fluctuations compared to social deprivation.

### DSEM Results

**Fig. 2:**
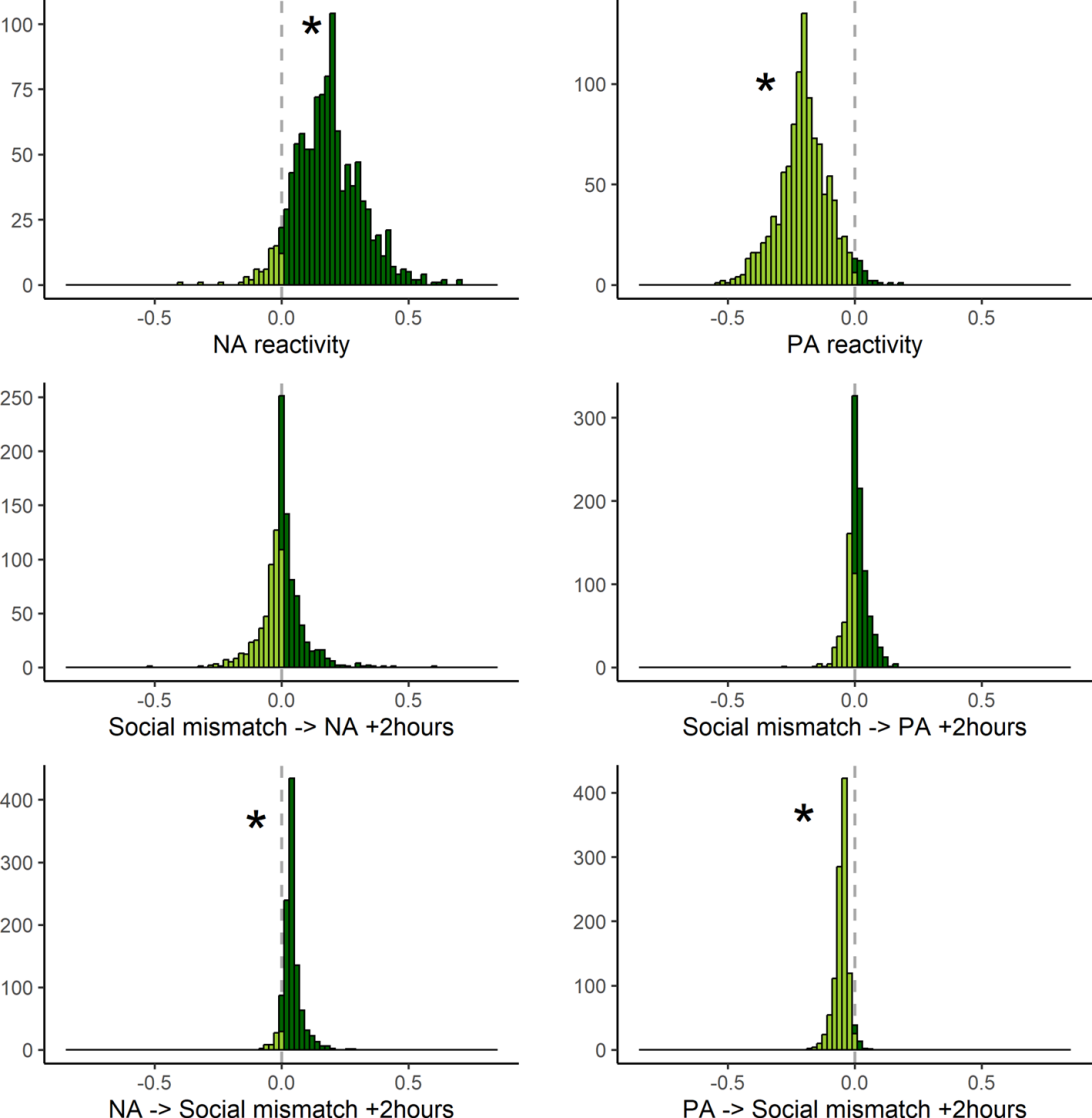
Individual differences in effects between social mismatch and negative and positive affect. For example, the histogram for NA reactivity shows that for most participants NA increases when there is more social mismatch, but participants differ in the strength of the effect. Similarly, the histogram for PA reactivity shows that for most participants PA decreases when there is more social mismatch. The star indicates significant variance between persons

On average across participants, the results of DSEM models showed that social mismatch (combining social deprivation and oversatiation) is related to more negative affect (β =.18) as well as less positive affect (β = -.19) at the same time point (see Table 3). There was no effect of social mismatch on either negative or positive affect two hours later (β =.00 and.01 respectively). However, both higher negative affect and lower positive affect were related to more social mismatch two hours later (β =.04 and -.05 respectively). The between-person variance indicated individual differences in most associations between social mismatch and negative and positive affect (see Fig. 2).

**Table 3:** DSEM results. Social mismatch, social deprivation, and social oversatiation

Social mismatch	Within-person average					Between-person variance			
**Negative affect**	Estimate	β	Post sd	95% CI		σ2	SD/Est	95% CI	
Reactivity (Mismatch—Affect)	0.08	**0.18**	0.01	0.17	0.19	**0.01**	0.13	0.01	0.01
Mismatch—> Affect + 2 h	0.00	0.00	0.01	−0.01	0.02	0.00	0.25	0.00	0.01
Affect—> Mismatch + 2 h	0.10	**0.04**	0.01	0.03	0.05	**0.05**	0.22	0.03	0.07
Affect—> Affect + 2 h	0.30	**0.30**	0.01	0.29	0.31	**0.04**	0.07	0.04	0.05
Mismatch—> Mismatch + 2 h	0.21	**0.21**	0.01	0.20	0.22	**0.04**	0.08	0.03	0.04
**Positive affect**	Estimate	β	Post sd	95% CI		σ2	SD/Est	95% CI	
Reactivity (Mismatch—Affect)	−0.10	**−0.19**	0.01	−0.21	−0.18	**0.01**	0.14	0.01	0.01
Mismatch—> Affect + 2 h	0.01	0.01	0.01	0.00	0.02	0.00	0.00	0.00	0.00
Affect—> Mismatch + 2 h	−0.11	**−0.05**	0.01	−0.06	−0.04	**0.02**	0.35	0.01	0.04
Affect—> Affect + 2 h	0.35	**0.35**	0.01	0.34	0.37	**0.03**	0.07	0.03	0.03
Mismatch—> Mismatch + 2 h	0.20	**0.20**	0.01	0.19	0.22	**0.04**	0.08	0.03	0.04
**Social deprivation**	***Within-person average***					***Between-person variance***			
**Negative affect**	Estimate	β	Post sd	95%CI		σ2	SD/Est	95%CI	
Reactivity (Mismatch—Affect)	0.02	**0.03**	0.01	0.01	0.06	**0.01**	0.09	0.01	0.01
Mismatch—> Affect + 2 h	0.01	0.02	0.01	−0.01	0.05	**0.03**	0.10	0.03	0.04
Affect—> Mismatch + 2 h	0.00	−0.01	0.02	−0.04	0.03	**0.26**	0.17	0.18	0.35
Affect—> Affect + 2 h	0.29	**0.29**	0.01	0.26	0.31	**0.07**	0.09	0.06	0.09
Mismatch—> Mismatch + 2 h	0.22	**0.22**	0.01	0.20	0.25	**0.08**	0.09	0.07	0.09
**Positive affect**	Estimate	β	Post sd	95% CI		σ2	SD/Est	95% CI	
Reactivity (Mismatch—Affect)	0.00	0.00	0.01	−0.02	0.03	**0.01**	0.20	0.01	0.01
Mismatch—> Affect + 2 h	0.01	0.01	0.01	−0.02	0.04	**0.01**	0.27	0.01	0.02
Affect—> Mismatch + 2 h	−0.03	−0.02	0.01	−0.04	0.01	**0.22**	0.17	0.16	0.29
Affect—> Affect + 2 h	0.38	**0.38**	0.01	0.35	0.40	**0.04**	0.11	0.04	0.05
Mismatch—> Mismatch + 2 h	0.23	**0.23**	0.01	0.20	0.25	**0.08**	0.08	0.07	0.10
**Social oversatiation**	***Within-person average***					***Between-person variance***			
**Negative affect**	Estimate	β	Post sd	95% CI		σ2	SD/Est	95% CI	
Reactivity (Mismatch—Affect)	0.13	**0.26**	0.01	0.24	0.28	**0.01**	0.07	0.01	0.02
Mismatch—> Affect + 2 h	−0.02	**−0.03**	0.01	−0.05	−0.01	**0.02**	0.08	0.02	0.03
Affect—> Mismatch + 2 h	0.13	**0.06**	0.01	0.04	0.08	**0.33**	0.10	0.27	0.40
Affect—> Affect + 2 h	0.29	**0.29**	0.01	0.28	0.31	**0.06**	0.08	0.06	0.07
Mismatch—> Mismatch + 2 h	0.20	**0.20**	0.01	0.18	0.22	**0.07**	0.08	0.06	0.08
**Positive affect**	Estimate	β	Post sd	95% CI		σ2	SD/Est	95% CI	
Reactivity (Mismatch—Affect)	−0.18	**−0.29**	0.01	−0.31	−0.27	**0.01**	0.09	0.01	0.01
Mismatch—> Affect + 2 h	0.01	0.01	0.01	−0.01	0.03	**0.01**	0.22	0.01	0.01
Affect—> Mismatch + 2 h	−0.14	**−0.08**	0.01	−0.10	−0.06	**0.23**	0.10	0.19	0.28
Affect—> Affect + 2 h	0.35	**0.35**	0.01	0.33	0.36	**0.04**	0.10	0.03	0.05
Mismatch—> Mismatch + 2 h	0.19	**0.19**	0.01	0.17	0.21	**0.07**	0.08	0.06	0.08

The bold beta's indicate significant effects

Differences emerged when dividing social mismatch into social deprivation and social oversatiation separately (see Table 3). Social deprivation was only related to higher negative affect (β =.03) at the same time point. Social oversatiation was related to more negative affect (β =.26) and less positive affect (β = -.29) at the same time point. Furthermore, both higher negative affect (β =.06) and lower positive affect (β = -.08) were related to more social oversatiation two hours later. Unexpectedly, social oversatiation was related to *less* negative affect two hours later (β = -.03).

### Individual Differences

Next, we investigated who is more influenced by social mismatch in their positive and negative affect. We ran regression models predicting the concurrent and cross-lagged associations between negative or positive affect and social mismatch. After correction for multiple testing, two associations remained significant (see Table 4). For younger versus older adults (β =.15), there is a stronger negative relation between social mismatch and positive affect at the same time point, i.e., a stronger reactivity to social stress in younger versus older people. The observed age effect was not part of our preregistered hypotheses and should therefore be interpreted as exploratory. Furthermore, people with a stronger genetic predisposition for wellbeing (β = -.16) show a stronger reactivity in their positive affect to social stress as well. The expected associations with loneliness and depressive symptoms were not found. When separating social mismatch in social deprivation and oversatiation, effects were similar, but no effects remained significant after correcting for multiple testing (see supplementary Table S1 and Figs S1 and S2).

**Table 4:** Individual differences regressions

Overall social mismatch	NA reactivity			NA—> Mismatch + 2 h			Mismatch—> NA + 2 h		
	Estimate	SE	p	Estimate	SE	p	Estimate	SE	p
(Intercept)	−0.08	0.10	0.457	0.09	0.10	0.345	0.04	0.08	0.668
Sex	0.22	0.10	*0.030*	−0.02	0.09	0.866	−0.09	0.08	0.281
Age during EMA	−0.10	0.05	0.068	−0.08	0.04	0.065	0.04	0.04	0.421
PGS loneliness	0.02	0.04	0.626	0.05	0.04	0.247	0.07	0.04	0.089
PGS wellbeing	0.06	0.05	0.278	−0.05	0.06	0.383	−0.04	0.05	0.417
PGS depressive symptoms	−0.02	0.05	0.656	0.06	0.05	0.211	−0.05	0.04	0.265
Loneliness	0.07	0.06	0.238	−0.06	0.06	0.275	−0.07	0.05	0.174
Depressive symptoms	0.08	0.06	0.173	0.08	0.06	0.145	0.13	0.05	*0.013*
Wellbeing	−0.01	0.05	0.910	−0.02	0.05	0.707	0.05	0.04	0.231
	**PA reactivity**			**PA—> Mismatch + 2 h**			**Mismatch—> PA + 2 h**		
	Estimate	SE	p	Estimate	SE	p	Estimate	SE	p
(Intercept)	0.04	0.10	0.672	0.01	0.08	0.944	−0.04	0.08	0.645
Sex	−0.16	0.10	0.102	−0.06	0.08	0.462	0.02	0.08	0.759
Age during EMA	**0.15**	0.05	**0.003**	0.10	0.04	*0.023*	−0.01	0.04	0.813
PGS loneliness	0.01	0.05	0.810	−0.06	0.04	0.129	−0.09	0.05	*0.049*
PGS wellbeing	**−0.16**	0.05	**0.002**	0.07	0.05	0.142	0.06	0.04	0.172
PGS depressive symptoms	−0.10	0.05	*0.033*	−0.05	0.04	0.292	0.06	0.05	0.215
Loneliness	−0.03	0.06	0.570	0.06	0.05	0.264	0.03	0.05	0.498
Depressive symptoms	−0.04	0.06	0.451	−0.15	0.05	*0.007*	−0.10	0.05	0.063
Wellbeing	0.02	0.06	0.753	−0.07	0.05	0.159	−0.09	0.05	0.053

*Note* Bold p-values indicate significant after False discovery rate (FDR)correction

## Discussion

Social contact is essential for wellbeing. However, not all social interactions are related to positive affect, as mismatches can arise between the desire for social connection and actual social environment. We investigated within- and between-person associations between social mismatch and affective wellbeing in daily life. Across seven days, people were more often with other people (63%) compared to alone (37%), although this differed widely among participants (range: 0–100). On average, being with others was associated with about 0.4 SD higher positive affect and 0.2 SD lower negative affect than being alone, indicating a small-to-moderate but consistent benefit of social contact. However, mismatches occurred. From time to time, participants indicated *social deprivation* (M = 3.67), characterized by the absence of desired social contact, or *social oversatiation* (M = 2.65), the experience of unwanted company or social contact. DSEM results showed that higher negative affect and lower positive affect were related to more social mismatch, both at the same time point and two hours later. The inverse cross-lagged relationship—social mismatch related to affect two hours later—was only observed for social oversatiation. Unexpectedly, social oversatiation was related to *less* negative affect two hours later. Part of the individual differences in the effects were explained by age and genetic predisposition for wellbeing.

### Social Mismatch

We showed affective reactivity to social mismatch, indicating that social mismatch is stressful for individuals and is related to their momentary positive and negative affect. This experienced stress is arises from the appraisal of the social context (Vaessen et al., 2023). When individuals experience more positive affect, it might be easier to perceive their social environment in a favourable light, even in challenging daily settings (e.g., crowded trains after a long day). Negative affect might intensify feelings of mismatch, turning social experiences into negative ones.

When exploring the effects of social deprivation and oversatiation separately, participants reported more frequent and stronger experiences of social deprivation (mean = 3.67) compared to social oversatiation (mean = 2.65). Although social deprivation was more common, the estimated associations of social oversatiation and affect were larger for negative affect (β =.26 vs. β =.03) and positive affect (β = −.29 vs. β ≈ 0) at the same time point. These findings partially replicate those of Krämer et al. (2023). In their smaller sample (N = 306) using two days of EMA, social oversatiation was associated with reduced positive affect and increased negative affect, while social deprivation showed no significant associations with affect. In our sample (N = 1086) with a longer EMA period (seven days), we observed similar patterns for social oversatiation, along with an effect of social deprivation on negative affect at the same time point. Overall, unwanted social interactions may thus be perceived as more stressful and have stronger effects, while social mismatch due to the absence of social contact might be less distressing.

The time frame matters for interpreting social processes. We additionally investigated the cross-lagged associations, 2 h apart. Higher positive affect (β =.04) and lower negative affect (β = −.05) were related to less subsequent social mismatch, suggesting that affective states potentially influence the ability or motivation of individuals to align social contexts with social needs. Affective wellbeing can thus act as a catalyst, with positive affect enhancing the likelihood of seeking out social environments that meet the current needs, whereas negative affect might impair the ability to navigate social contexts effectively. As these effects seemed to mainly be driven by social oversatiation, these findings align with the idea that good affective wellbeing is a prerequisite for engaging in social interactions (Elmer, 2021; Liu et al., 2021).

The other way around, overall social mismatch was not related to positive or negative affect two hours later (β =.00 and.01), suggesting that social stress does not have a lasting effect on wellbeing. However, social oversatiation specifically was related to negative affect two hours later, but unexpectedly, more oversatiation was related to *less* negative affect two hours later. The effect was small (β = –0.07), but suggests that although social contact can be rated as unwanted, the associations with wellbeing a few hours later are positive. If causal, a potential mechanism could be the prevention of later feelings of loneliness by the (undesired) interaction, resulting in less subsequent negative affect. These results reflect the complex nature of social needs and interactions; short-term discomfort in social contact could be balanced by longer-term emotional benefits (Umberson & Karas Montez, 2010).

### Individual Differences

Individual differences in associations between social mismatch and positive and negative affect could be partly explained by other variables. Bivariate correlations showed that spending more time alone and experiencing more social mismatch, particularly social oversatiation, was associated with higher levels of loneliness, greater depressive symptoms, and lower overall wellbeing, as we expected. The association of social oversatiation and loneliness confirms that loneliness is about the subjective feeling and not the presence of others (Perlman & Peplau, 1981)*.* In regression models including all predictors, two effects remained significant after correcting for multiple testing. Whereas the expected associations with loneliness and depressive symptoms were not found, individuals with a stronger genetic predisposition for wellbeing showed stronger positive affective reactivity to social mismatch. This suggests that those individuals could be more sensitive to changes in their social environment, potentially due to an inherent focus on wellbeing and social contact. Genetic predispositions may thus shape how individuals experience and react to their social surroundings (Assary et al., 2024), influencing the lens through which daily social interactions and needs are interpreted.

Furthermore, younger adults showed stronger positive affective reactivity to social mismatch compared to older adults, with the difference corresponding to 0.15 SD lower positive affect, suggesting that younger people are more sensitive to discrepancies between their social context and desires. This age effect was not part of our preregistered hypotheses and should therefore be interpreted as exploratory. However, it is in line with previous findings of adolescence and young adulthood as a period of increased sensitivity to social context and peer interactions (Lam et al., 2014; Orben et al., 2020; Seiffge-Krenke et al., 2009) and large rewards of social interactions in adolescence (Altikulaç et al., 2019). Although these effects are modest in size, they may be practically meaningful given the frequency of social encounters in everyday life.

### Limitations and Future Directions

The social context variables in this study captured whether participants were with others or alone, without specifying whether they had direct interactions or the specific social partner. This could have influenced the results, as simply being near others does not guarantee meaningful social engagement. This limitation is especially relevant for social oversatiation, which may depend strongly on the quality of the interaction (e.g., closeness, enjoyment, perceived obligation) and on the identity of the social partner. Future research should include more detailed information about social partner characteristics and interaction quality to better understand when and with whom social contact becomes beneficial or overwhelming.. Individual characteristics are important as well. For example, while lonely individuals tend to experience more negative affect during social interactions compared to less lonely individuals (Hawkley et al., 2023), other studies have found that lonely individuals benefit more from social contact—especially when it involves close relationships—than those who are less lonely (Tang et al., 2022; van Roekel et al., 2018). Another direction for future research is the temporal proximity between measurements. We used a two-hour interval, which captures the relatively short-term effects. Future research could explore varying time intervals to better understand the timing of affective responses to social experiences.

Finally, although we identified individual differences in affective responses to social mismatch, we only explained a small portion of the variance. Future research could investigate additional factors—personality traits, coping strategies, or contextual influences—that may contribute to the individual differences.

Although the associations of social mismatch on affect were modest in this study, they can be meaningful because being alone and social encounters occur many times a day and effects can accumulate. Our findings indicate that interventions and public health strategies should move beyond simply increasing social contact to optimising the fit between the personal characteristics, social needs and social contexts, for example by supporting skills to recognise preferred social interactions and manage social demands. The small but unexpected lagged effect of oversatiation on lower negative affect also hints that brief unwanted contact may protect against later loneliness; although longitudinal work is needed to confirm this mechanism. Finally, individuals who show stronger reactivity may be a particularly relevant target group for just-in-time interventions or training in recognising and acting on their momentary social needs.

## Conclusion

Our results highlight the complex relationship between social contact, social mismatch, and affective wellbeing. While being with others is related to higher positive affect and lower negative affect, mismatches between social desires and the actual social context occur. This mismatch—especially social oversatiation—is related to momentary wellbeing, although not always negatively. Furthermore, the findings suggest that affective states influence the ability or motivation of individuals to align social contexts with social needs, i.e., affective wellbeing can act as a catalyst. Individual differences, including age and wellbeing genetic predisposition, influence how people react to social mismatch. These findings emphasize the importance of considering both social context and individual characteristics when assessing affective wellbeing and the importance of personal and contextual factors when designing social interventions to improve wellbeing and mental health.

## Supplementary Information

Below is the link to the electronic supplementary material.

Supplementary file1 (DOCX 258 KB)

## Data Availability

The data that support the findings of this study can be requested via the Netherlands Twin Register (https://ntr-data-request.psy.vu.nl/).

## Declarations

### Preregistration

The study was preregistered (https://osf.io/r2x3u).

### Analysis Scripts

All analysis scripts are publicly available (https://osf.io/drtah/).

## References

[CR1] Abdellaoui, A., Hottenga, J. J., Knijff, P. D., et al. (2013). Population structure, migration, and diversifying selection in the Netherlands. *European Journal of Human Genetics,**21*, 1277–1285. 10.1038/ejhg.2013.48 23531865 10.1038/ejhg.2013.48 PMC3798851

[CR2] Achenbach, T. M., & Rescorla, L. A. (2003). *Manual for the ASEBA adult forms & profiles*. University of Vermont, Research Center for Children, Youth, & Families.

[CR3] Altikulaç, S., Bos, M. G. N., Foulkes, L., et al. (2019). Age and gender effects in sensitivity to social rewards in adolescents and young adults. *Frontiers in Behavioral Neuroscience,**13*, Article 456625. 10.3389/FNBEH.2019.00171/BIBTEX 10.3389/fnbeh.2019.00171 PMC6681770 31417377

[CR4] American Psychiatric Association (2013). Diagnostic and statistical manual of mental disorders, fifth edition [DSM-5]

[CR5] Asparouhov, T., Hamaker, E. L., & Muthén, B. (2018). Dynamic structural equation models. *Structural Equation Modeling: A Multidisciplinary Journal,**25*, 359–388. 10.1080/10705511.2017.1406803

[CR6] Assary, E., Oginni, O. A., Morneau-Vaillancourt, G., et al. (2024). Genetics of environmental sensitivity and its association with variations in emotional problems, autistic traits, and wellbeing. *Molecular Psychiatry*. 10.1038/S41380-024-02508-6 10.1038/s41380-024-02508-6 PMC11412899 38499655

[CR7] Baselmans, B. M. L., Jansen, R., Ip, H. F., et al. (2019). Multivariate genome-wide analyses of the well-being spectrum. *Nature Genetics,**51*, 445–451. 10.1038/s41588-018-0320-8 30643256 10.1038/s41588-018-0320-8

[CR67] Benjamini, Y., & Hochberg, Y. (1995). Controlling the false discovery rate: a practical and powerful approach to multiple testing. *Journal of the Royal statistical society: series B (Methodological)*, 57(1), 289-300. 10.1111/j.2517-6161.1995.tb02031.x

[CR8] Bernstein, M. J., Zawadzki, M. J., Juth, V., et al. (2018). Social interactions in daily life: Within-person associations between momentary social experiences and psychological and physical health indicators. *Journal of Social and Personal Relationships,**35*, 372–394. 10.1177/0265407517691366

[CR11] Bülow, A., Boele, S., & Keijsers, L. (2020). *One hundred days of my life from the ADAPT project: Assessing the Dynamics between Parenting and Adaptation in Teens*. Retrieved from osf.io/svyau.

[CR9] Booij, S. H., Snippe, E., Jeronimus, B. F., et al. (2018). Affective reactivity to daily life stress: Relationship to positive psychotic and depressive symptoms in a general population sample. *Journal Of Affective Disorders,**225*, 474–481. 10.1016/J.JAD.2017.08.051 28863300 10.1016/j.jad.2017.08.051

[CR10] Bralten, J., Mota, N. R., Klemann, C. J. H. M., et al. (2021). Genetic underpinnings of sociability in the general population. *Neuropsychopharmacology,**46*, 1627. 10.1038/S41386-021-01044-Z 34054130 10.1038/s41386-021-01044-z PMC8280100

[CR12] Cacioppo, J. T., & Patrick, W. (2008). *Loneliness: Human Nature and the Need for Social Connection.*

[CR13] Cohen, L. H., Gunthert, K. C., Butler, A. C., et al. (2005). Daily affective reactivity as a prospective predictor of depressive symptoms. *Journal of Personality,**73*, 1687–1714. 10.1111/J.0022-3506.2005.00363.X 16274450 10.1111/j.0022-3506.2005.00363.x

[CR14] Coplan, R. J., Hipson, W. E., Archbell, K. A., et al. (2019). Seeking more solitude: Conceptualization, assessment, and implications of aloneliness. *Personality and Individual Differences,**148*, 17–26. 10.1016/J.PAID.2019.05.020

[CR15] Day, F. R., & Ong, K. K. (2018). Perry JRB (2018) Elucidating the genetic basis of social interaction and isolation. *Nature Communications,**91*(9), 1–6. 10.1038/s41467-018-04930-1 10.1038/s41467-018-04930-1 PMC6030100 29970889

[CR16] de Vries LP, Bartels M 2025 Individual differences in wellbeing dynamics: a genetically-informed comparison of Ecological Momentary Assessment and longitudinal survey data. * Personality and Individual Differences, 236,* 112996. 10.1016/j.paid.2024.112996

[CR17] Diener, E., Emmons, R. A., Larsen, R. J., & Griffin, S. (1985). The satisfaction with life scale. *Journal of Personality Assessment,**49*, 71–75. 10.1207/s15327752jpa4901_13 16367493 10.1207/s15327752jpa4901_13

[CR18] Distel, M. A., Rebollo-Mesa, I., & Abdellaoui, A. (2010). Familial resemblance for loneliness. *Behavior Genetics*. 10.1007/s10519-010-9341-5 10.1007/s10519-010-9341-5 PMC2886905 20145989

[CR19] Elmer, T. (2021). In which direction does happiness predict subsequent social interactions? a commentary on quoidbach et al. (2019). *Psychol Sci*, *32*, 955–959. 10.1177/0956797620956981/SUPPL_FILE/SJ-DOCX-1-PSS-10.1177_0956797620956981.DOCX 10.1177/0956797620956981 33950713

[CR20] Flint, J. (2023). The genetic basis of major depressive disorder. *Molecular Psychiatry*(6). 10.1038/s41380-023-01957-9 10.1038/s41380-023-01957-9 PMC10611584 36702864

[CR21] Gariépy, G., Honkaniemi, H., & Quesnel-Vallée, A. (2016). Social support and protection from depression: Systematic review of current findings in Western countries. *British Journal of Psychiatry,**209*, 284–293. 10.1192/BJP.BP.115.169094 10.1192/bjp.bp.115.169094 27445355

[CR22] Hallquist, M. N., & Wiley, J. F. (2018). MplusAutomation: An R package for facilitating large-scale latent variable analyses in Mplus. *Structural Equation Modeling: A Multidisciplinary Journal,**25*, 621–638. 10.1080/10705511.2017.1402334 30083048 10.1080/10705511.2017.1402334 PMC6075832

[CR23] Hamaker, E. L., Asparouhov, T., Brose, A., et al. (2018). At the frontiers of modeling intensive longitudinal data: Dynamic structural equation models for the affective measurements from the COGITO study. *Multivariate Behavioral Research,**53*, 820–841. 10.1080/00273171.2018.1446819 29624092 10.1080/00273171.2018.1446819

[CR24] Hawkley, L. C., Preacher, K.J., Cacioppo John, T. (2023). Multilevel modeling of social interactions and mood in lonely and socially connected individuals: The MacArthur Social Neuroscience Studies. In: *Oxford Handbook of Methods in Positive Psychology*

[CR25] Houben, M., Van Den Noortgate, W., & Kuppens, P. (2015). The relation between short-term emotion dynamics and psychological well-being: A meta-analysis. *Psychological Bulletin*. 10.1037/a0038822 10.1037/a0038822 25822133

[CR26] Howard, D. M., Adams, M. J., Clarke, T. K., et al. (2019). Genome-wide meta-analysis of depression identifies 102 independent variants and highlights the importance of the prefrontal brain regions. *Nature Neuroscience*. 10.1038/s41593-018-0326-7 10.1038/s41593-018-0326-7 PMC6522363 30718901

[CR27] Hughes, M. E., Waite, L..J., Hawkley, L. C., & Cacioppo, J. T. (2004). A short scale for measuring loneliness in large surveys: Results from two population-based studies. Res. Aging 26 10.1177/0164027504268574 PMC2394670 18504506

[CR28] Keyes, C. L. M. (1998). *Social Well-Being. Soc Psychol Q,**61*, 121–140.

[CR29] Krämer, M. D., Roos, Y., Schoedel, R., et al. (2023). Social dynamics and affect: Investigating within-person associations in daily life using experience sampling and mobile sensing. *Emotion*. 10.1037/EMO0001309 10.1037/emo0001309 37917503

[CR30] Kuppens, P., & Verduyn, P. (2017). Emotion dynamics. *Current Opinion in Psychology,**17*, 22–26. 10.1016/J.COPSYC.2017.06.004 28950968 10.1016/j.copsyc.2017.06.004

[CR31] Lai, M. H. C. (2021). Composite reliability of multilevel data: It’s about observed scores and construct meanings. *Psychological Methods,**26*, 90–102. 10.1037/MET0000287 32673041 10.1037/met0000287

[CR33] Lambert, L., Lomas, T., Weijer, M. P. va. de, et al. (2020). Towards a greater global understanding of wellbeing: A proposal for a more inclusive measure. Int J Wellbeing 10:. 10.5502/ijw.v10i2.1037

[CR32] Lam, C. B., Mchale, S. M., & Crouter, A. C. (2014). Time with peers from middle childhood to late adolescence: Developmental course and adjustment correlates. *Child Development,**85*, 1677–1693. 10.1111/CDEV.12235 24673293 10.1111/cdev.12235 PMC4107039

[CR34] Lay, J. C., Pauly, T., Graf, P., et al. (2019). By myself and liking it? Predictors of distinct types of solitude experiences in daily life. *Journal of Personality,**87*, 633–647. 10.1111/JOPY.12421 30003553 10.1111/jopy.12421

[CR35] Lazarus, R. S., & Folkman, S. (1984). *Stress, appraisal, and coping*. Springer Publishing Company.

[CR36] Leger, K. A., Charles, S. T., & Almeida, D. M. (2018). Let it go: Lingering negative affect in response to daily stressors is associated with physical health years later. *Psychological Science,**29*, 1283–1290. 10.1177/0956797618763097 29553880 10.1177/0956797618763097 PMC6088503

[CR37] Li, G., Li, Y., Lam, A. I. F., et al. (2023). Understanding the protective effect of social support on depression symptomatology from a longitudinal network perspective. *BMJ Mental Health,**26*, 1–7. 10.1136/BMJMENT-2023-300802 10.1136/bmjment-2023-300802 PMC10689368 38030405

[CR38] Ligthart, L., van Beijsterveldt, C. E. M., Kevenaar, S. T., et al. (2019). The Netherlands twin register: Longitudinal research based on twin and twin-family designs. *Twin Research and Human Genetics,**22*, 623–636. 10.1017/thg.2019.93 31666148 10.1017/thg.2019.93

[CR40] Liu, H., Fang, B., Li, Y., & Lou, V. W. Q. (2021). Initially negative affect predicts lower satisfaction with future social contact: A time-lagged analysis using ecological momentary assessment. *The Journals of Gerontology. Series B, Psychological Sciences and Social Sciences,**76*, 295–305. 10.1093/GERONB/GBAA024 32060519 10.1093/geronb/gbaa024

[CR39] Liu, H., Xie, Q. W., & Lou, V. W. Q. (2019). Everyday social interactions and intra-individual variability in affect: A systematic review and meta-analysis of ecological momentary assessment studies. *Motivation and Emotion,**43*, 339–353. 10.1007/S11031-018-9735-X/METRICS

[CR41] Lomas, T., Bartels, M., Van De Weijer, M., et al. (2022). The architecture of happiness. *Emotion Review,**14*, 288–309. 10.1177/17540739221114109

[CR42] Minică, C. C., Dolan, C. V., Kampert, M. M. D., et al. (2015). Sandwich corrected standard errors in family-based genome-wide association studies. *European Journal of Human Genetics,**23*, 388. 10.1038/ejhg.2014.94 24916646 10.1038/ejhg.2014.94 PMC4326721

[CR43] Monninger, M., Aggensteiner, P. M., Pollok, T. M., et al (2022) Real-time individual benefit from social interactions before and during the lockdown: The crucial role of personality, neurobiology and genes. *Transl Psychiatry*, *121*(12), 1–10. 10.1038/s41398-022-01799-z 10.1038/s41398-022-01799-z PMC8777449 35064105

[CR66] Neale Lab data base (2020). Retrieved from: https://www.nealelab.is/uk-biobank

[CR44] Okbay, A., Baselmans, B. M. L., De Neve, J.-E., et al. (2016). Genetic variants associated with subjective well-being, depressive symptoms and neuroticism identified through genome-wide analyses. *Nature Genetics,**48*, 624–633. 10.1038/ng.3552 27089181 10.1038/ng.3552 PMC4884152

[CR45] Orben, A., Tomova, L., & Blakemore, S. J. (2020). The effects of social deprivation on adolescent development and mental health. *The Lancet Child & Adolescent Health,**4*, 634–640. 10.1016/S2352-4642(20)30186-3 32540024 10.1016/S2352-4642(20)30186-3 PMC7292584

[CR46] Perlman, D., & Peplau, L. (1981). Toward a social psychology of loneliness.*Pers Relatsh**3*.

[CR47] Pinquart, M., & Sörensen, S. (2000). Influences of socioeconomic status, social network, and competence on subjective well-being in later life: A meta-analysis. *Psychology and Aging,**15*, 187–224. 10.1037/0882-7974.15.2.187 10879576 10.1037//0882-7974.15.2.187

[CR48] Purcell, S. M., Wray, N. R., Stone, J. L., et al. (2009). Common polygenic variation contributes to risk of schizophrenia and bipolar disorder. *Nature,**460*, 748–752. 10.1038/nature08185 19571811 10.1038/nature08185 PMC3912837

[CR49] Reininghaus, U., Kempton, M. J., Valmaggia, L., et al. (2016). Stress sensitivity, aberrant salience, and threat anticipation in early psychosis: An experience sampling study. *Schizophrenia Bulletin,**42*, 712–722. 10.1093/SCHBUL/SBV190 26834027 10.1093/schbul/sbv190 PMC4838104

[CR50] Ren, D., Stavrova, O., & Loh, W. W. (2022). Nonlinear effect of social interaction quantity on psychological well-being: Diminishing returns or inverted u? *Journal of Personality and Social Psychology,**122*, 1056–1074. 10.1037/PSPI0000373 34591543 10.1037/pspi0000373

[CR51] Rico-Uribe, L. A., Caballero, F. F., Martín-María, N., et al. (2018). Association of loneliness with all-cause mortality: A meta-analysis. *PLoS One,**13*, Article e0190033. 10.1371/JOURNAL.PONE.0190033 29300743 10.1371/journal.pone.0190033 PMC5754055

[CR52] Seiffge-Krenke, I., Aunola, K., & Nurmi, J. E. (2009). Changes in stress perception and coping during adolescence: The role of situational and personal factors. *Child Development,**80*, 259–279. 10.1111/J.1467-8624.2008.01258.X 19236405 10.1111/j.1467-8624.2008.01258.x

[CR53] Socrates, A., Mullins, N., Gur, R., et al. (2023). *Polygenic risk of Social-isolation and its influence on social behavior, psychosis, depression and autism spectrum disorder*. Res Sq.10.21203/RS.3.RS-2583059/V1

[CR54] Stone, A. A., & Shiffman, S. (1994). Ecological momentary assessment (EMA) in behavorial medicine. *Annals of Behavioral Medicine,**16*, 199–202.

[CR55] Sun, J., Harris, K., & Vazire, S. (2020). Is well-being associated with the quantity and quality of social interactions? *Journal of Personality and Social Psychology,**119*, 1478–1496. 10.1037/PSPP0000272 31647273 10.1037/pspp0000272

[CR56] Tang, W. C., yin, Wong CS man, Wong T yat, et al. (2022). Social context and loneliness in an epidemiological youth sample using the Experience Sampling Method. *J Psychiatr Res,**156*, 429–436. 10.1016/J.JPSYCHIRES.2022.10.041 36323146 10.1016/j.jpsychires.2022.10.041

[CR57] Umberson, D., & Karas Montez, J. (2010). Social relationships and health: A flashpoint for health policy. *Journal of Health and Social Behavior,**51*, S54–S66. 10.1177/0022146510383501 20943583 10.1177/0022146510383501 PMC3150158

[CR58] Vaessen, T., Reininghaus, U., & Myin-Germeys, I. (2023). Stress Assessment in Daily Life Using the Experience Sampling Method. *Palgrave Handb Occup Stress,*117–136. 10.1007/978-3-031-27349-0_7/FIGURES/1

[CR59] van de Weijer, M. P., de Vries, L. P., & Bartels, M. (2022). Happiness and well-being: The value and findings from genetic studies. In A. Tarnoki, D. Tarnoki, J. Harris, & N. Segal (Eds.), *Twin Research for Everyone* (pp. 295–322). Academic Press.

[CR60] van Roekel, E., Verhagen, M., Engels, R. C. M. E., et al. (2018). Trait and state levels of loneliness in early and late adolescents: Examining the differential reactivity hypothesis. *Journal of Clinical Child & Adolescent Psychology,**47*, 888–899. 10.1080/15374416.2016.1146993 27191708 10.1080/15374416.2016.1146993

[CR61] Watson, D., Clark, L. A., & Tellegen, A. (1988). Development and validation of brief measures of positive and negative affect: The PANAS scales. *Journal of Personality and Social Psychology,**54*, 1063–1070. 10.1037/0022-3514.54.6.1063 3397865 10.1037//0022-3514.54.6.1063

[CR62] Wheeler, L., Reis, H., & Nezlek, J. B. (1983). Loneliness, social interaction, and sex roles. *Journal of Personality and Social Psychology*. 10.1037/0022-3514.45.4.943 10.1037//0022-3514.45.4.943 6631669

[CR63] Wray, N. R., Goddard, M. E., & Visscher, P. M. (2007). Prediction of individual genetic risk to disease from genome-wide association studies. *Genome Research,**17*, 1520–1528. 10.1101/gr.6665407 17785532 10.1101/gr.6665407 PMC1987352

[CR64] Wrzus, C., Roos, Y., Krämer, M. D. , & Richter, D. (2024). Individual differences in short-term social dynamics: Theoretical perspective and empirical development of the social dynamics scale. * Curr Psychol,* 1–21. 10.1007/S12144-024-05868-Y/FIGURES/5

