# Supplementary material for: Social Mismatch and Affective Wellbeing: An Ecological Momentary Assessment Study
Source: J Happiness Stud. 2025 Oct 23;26(8):136. doi: 10.1007/s10902-025-00965-6 (PMC12549768; doi:10.1007/s10902-025-00965-6)
Supplement: Supplementary file 1 — Supplementary file1 (DOCX 258 KB) [file 10902_2025_965_MOESM1_ESM.docx]

**Supplementary Material**


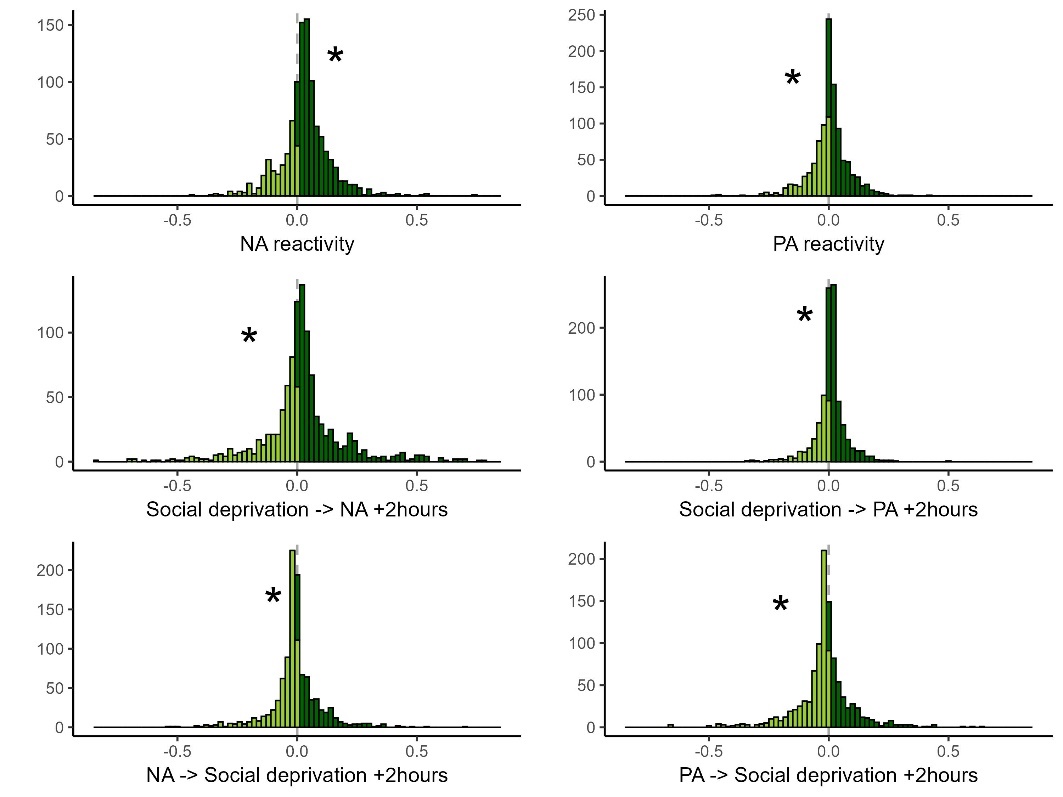


**Figure S1.** Individual differences in the associations between negative and positive affect and social deprivation.


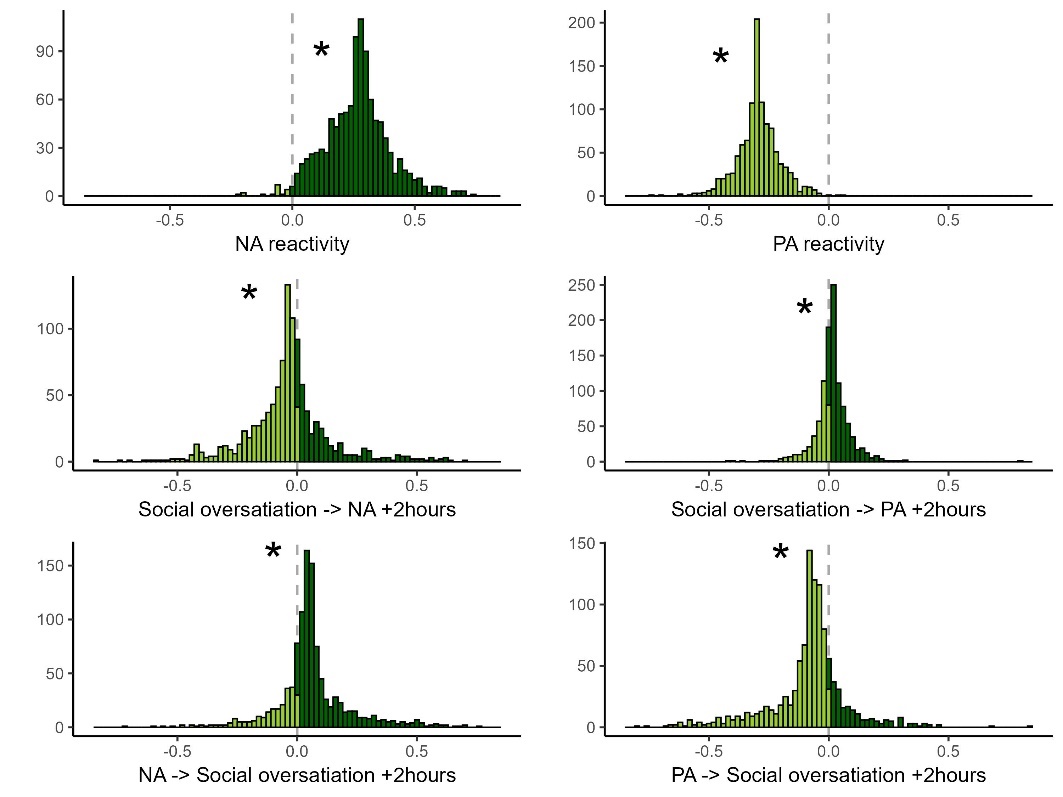


**Figure S2.** Individual differences in the associations between negative and positive affect and social oversatiation.

**Table S1.** Individual differences in the associations between negative and positive affect and social deprivation and oversatiation**.**

| **Social deprivation** | **NA reactivity** | | | **NA -> Mismatch +2h** | | | **Mismatch -> NA +2h** | | |
| --- | --- | --- | --- | --- | --- | --- | --- | --- | --- |
|  | Estimate | SE | p | Estimate | SE | p | Estimate | SE | p |
| (Intercept) | -0.08 | 0.08 | 0.313 | 0.08 | 0.07 | 0.293 | -0.07 | 0.09 | 0.421 |
| Sex | 0.23 | 0.09 | 0.008 | -0.16 | 0.08 | 0.034 | 0.02 | 0.09 | 0.819 |
| Age | -0.10 | 0.05 | 0.043 | 0.03 | 0.04 | 0.431 | 0.05 | 0.05 | 0.376 |
| PGS loneliness | 0.02 | 0.04 | 0.725 | 0.03 | 0.04 | 0.441 | -0.03 | 0.06 | 0.549 |
| PGS wellbeing | 0.06 | 0.05 | 0.284 | 0.01 | 0.05 | 0.777 | 0.06 | 0.06 | 0.337 |
| PGS depressive symptoms | -0.02 | 0.05 | 0.743 | 0.10 | 0.05 | 0.026 | -0.04 | 0.05 | 0.439 |
| Loneliness | -0.03 | 0.06 | 0.560 | 0.08 | 0.06 | 0.181 | -0.10 | 0.06 | 0.099 |
| Depressive symptoms | 0.01 | 0.05 | 0.921 | -0.04 | 0.05 | 0.479 | 0.16 | 0.06 | 0.006 |
| Wellbeing | -0.08 | 0.05 | 0.118 | 0.02 | 0.05 | 0.712 | 0.08 | 0.05 | 0.134 |
|  | **PA reactivity** | | | **PA -> Mismatch +2h** | | | **Mismatch -> PA +2h** | | |
|  | Estimate | SE | p | Estimate | SE | p | Estimate | SE | p |
| (Intercept) | 0.02 | 0.09 | 0.860 | -0.16 | 0.09 | 0.068 | 0.09 | 0.08 | 0.290 |
| Sex | -0.13 | 0.09 | 0.141 | 0.12 | 0.08 | 0.134 | -0.03 | 0.08 | 0.671 |
| Age | 0.09 | 0.05 | 0.084 | 0.01 | 0.05 | 0.799 | 0.00 | 0.05 | 0.980 |
| PGS loneliness | 0.00 | 0.05 | 0.996 | 0.01 | 0.04 | 0.790 | -0.08 | 0.04 | 0.060 |
| PGS wellbeing | -0.10 | 0.06 | 0.070 | 0.03 | 0.05 | 0.549 | 0.01 | 0.04 | 0.879 |
| PGS depressive symptoms | -0.07 | 0.05 | 0.199 | -0.06 | 0.05 | 0.182 | 0.02 | 0.05 | 0.660 |
| Loneliness | 0.01 | 0.07 | 0.886 | -0.01 | 0.06 | 0.918 | 0.02 | 0.06 | 0.711 |
| Depressive symptoms | 0.04 | 0.06 | 0.488 | -0.04 | 0.05 | 0.366 | -0.12 | 0.05 | 0.015 |
| Wellbeing | 0.14 | 0.06 | 0.028 | -0.09 | 0.05 | 0.086 | -0.07 | 0.05 | 0.173 |
| **Social oversatiation** | **NA reactivity** | |  | **NA -> Mismatch +2h** | | | **Mismatch -> NA +2h** | | |
|  | Estimate | SE | p | Estimate | SE | p | Estimate | SE | p |
| (Intercept) | -0.02 | 0.10 | 0.857 | 0.01 | 0.09 | 0.943 | -0.11 | 0.09 | 0.222 |
| Sex | 0.03 | 0.10 | 0.799 | 0.02 | 0.09 | 0.794 | 0.04 | 0.09 | 0.640 |
| Age | 0.02 | 0.05 | 0.673 | 0.03 | 0.04 | 0.462 | -0.02 | 0.05 | 0.646 |
| PGS loneliness | -0.07 | 0.05 | 0.151 | 0.04 | 0.04 | 0.313 | 0.04 | 0.05 | 0.413 |
| PGS wellbeing | 0.04 | 0.05 | 0.416 | -0.01 | 0.05 | 0.872 | -0.02 | 0.05 | 0.733 |
| PGS depressive symptoms | 0.02 | 0.05 | 0.758 | 0.01 | 0.05 | 0.804 | -0.10 | 0.05 | 0.040 |
| Loneliness | 0.12 | 0.06 | 0.037 | -0.02 | 0.06 | 0.727 | -0.04 | 0.05 | 0.481 |
| Depressive symptoms | -0.06 | 0.07 | 0.380 | 0.10 | 0.06 | 0.102 | -0.01 | 0.05 | 0.783 |
| Wellbeing | 0.04 | 0.05 | 0.504 | -0.07 | 0.05 | 0.199 | 0.02 | 0.05 | 0.744 |
|  | **PA reactivity** | |  | **PA -> Mismatch +2h** | | | **Mismatch -> PA +2h** | | |
|  | Estimate | SE | p | Estimate | SE | p | Estimate | SE | p |
| (Intercept) | -0.06 | 0.08 | 0.486 | 0.08 | 0.09 | 0.350 | 0.08 | 0.08 | 0.302 |
| Sex | 0.07 | 0.09 | 0.402 | -0.05 | 0.09 | 0.609 | -0.16 | 0.08 | 0.043 |
| Age | 0.06 | 0.05 | 0.211 | -0.03 | 0.04 | 0.436 | 0.06 | 0.04 | 0.192 |
| PGS loneliness | 0.06 | 0.05 | 0.250 | -0.06 | 0.05 | 0.168 | 0.00 | 0.05 | 0.947 |
| PGS wellbeing | -0.10 | 0.05 | 0.056 | 0.06 | 0.06 | 0.271 | 0.02 | 0.05 | 0.636 |
| PGS depressive symptoms | -0.09 | 0.05 | 0.092 | 0.03 | 0.05 | 0.604 | 0.07 | 0.04 | 0.067 |
| Loneliness | -0.12 | 0.05 | 0.025 | 0.05 | 0.06 | 0.451 | 0.06 | 0.05 | 0.232 |
| Depressive symptoms | 0.07 | 0.05 | 0.172 | -0.12 | 0.06 | 0.053 | 0.04 | 0.06 | 0.483 |
| Wellbeing | -0.01 | 0.05 | 0.761 | 0.02 | 0.05 | 0.747 | 0.00 | 0.05 | 0.974 |
